# Supplementary material for: Cloud BioLinux: pre-configured and on-demand bioinformatics computing for the genomics community
Source: BMC Bioinformatics. 2012 Mar 19;13:42. doi: 10.1186/1471-2105-13-42 (PMC3372431; doi:10.1186/1471-2105-13-42)
Supplement: Additional file 1 — Supplementary 1 Cloud BioLinux software documentation in the form of a mini, self-contained website. Users need to download and uncompress the .zip file, and open through a web browser the "index.html" file available on the main directory. (ZIP 1823 kb). [file 1471-2105-13-42-S1.ZIP › Cloud-BioLinux-Package-Documentation/Alignment.html]

Bio-Linux Software Documentation Pages

Back to search form

### Alignment

|  |  |
| --- | --- |
| Category | Applications |
| Alignment | FastTree    dialign |
| SubCategory | Applications |
| Consensus | cons    consambig    gap4    megamerger    merger    spin |
| Differences | act    diffseq |
| Dot\_plots | dotmatcher    dotpath    dotter    dottup    polydot |
| Editing | clcsequenceviewer    jalview    squint |
| Global | est2genome    fasta    ggsearch    glsearch    needle    needleall    stretcher    swat |
| Graphical | blixem    clcsequenceviewer    clustalx    dotter    jalview    jprofilegrid    mummerplot    nucmer2xfig    seaview    squint |
| arb    bl2seq    bwa    cross\_match    dba    fasta    fastf    fastm    fasts    fastx    fasty    flalign    glsearch    lalign    lfasta    matcher    plalign    plfasta    prenuc    prfx    prss    psw    pswdb    seqmatchall    sibsim4    sim4    ssearch    supermatcher    swat    tfastf    tfastm    tfasts    tfastx    tfasty    water    wordfinder    wordmatch |  |
| Multiple | annotate    clustalw    clustalx    combineMUMs    dialign    edialign    emma    exact-tandems    gaps    hmmalign    infoalign    jalview    lalign2list    mapview    mgaps    mocca    mummer    mummerplot    muscle    nucmer    nucmer2xfig    plotcon    postnuc    postpro    prank    prepro    prettyplot    PROmer    repeat-match    run-mummer1    run-mummer3    seaview    show-aligns    show-coords    show-tiling    showalign    squint    t-coffee    tranalign    yamap |
| Profiles | pfmake    pfsearch    pftools    psa2msa    ptof    ptoh |
| Statistics | alistat    pfscale    prfx    prss    weight |
